# Polystyrene microsphere and 5-fluorouracil release from custom-designed wound dressing films

**DOI:** 10.1186/2194-0517-2-1

**Published:** 2013-01-24

**Authors:** Maryam Mobed-Miremadi, Raki Komarla Nagendra, Sujana Lakshmi Ramachandruni, Jason James Rook, Mallika Keralapura, Michel Goedert

**Affiliations:** 1 Department of Biomedical, Chemical and Materials Engineering, San Jose State University, San Jose, CA 95192-0082 USA; 2 MSE Biomedical Devices Concentration, San Jose State University, San Jose, CA USA; 3 MSE Biomedical Engineering, San Jose State University, San Jose, CA USA; 4 Department of Electrical Engineering, San Jose State University, San Jose, CA USA

**Keywords:** Chitosan, Alginate, Microsphere, 5-FU, Pore size, Atomic force microscopy, Release modeling

## Abstract

Custom-designed wound dressing films of chitosan and alginate have been prepared by a casting/solvent evaporation method for hydrophobic therapeutic agent encapsulation. In this parametric study, the propylene glycol (PG) and calcium chloride (CaCl_2_) concentrations were varied for chitosan and alginate films, respectively. Mechanical and chemical inter-related responses under observations included thickness (*th*), elasticity *(E),* tensile strength (*TS*), sorption ability (S%) and kinetics of *in-vitro* drug release, specifically in terms of membrane time to burst (*t*_*B*_) and duration of release (*t*_*R*_). As shown by results of a one tailed t-test significance testing at the 95% confidence interval (α = 0.05), alginate films were significantly more elastic (p = 0.003), thinner (p = 0.004) and more susceptible to osmotic burst (p = 0.011) and characterized by a longer duration of release (p = 0.03). Meanwhile chitosan films exhibited superior moisture permeability (p = 0.006) and sorption characteristics (p = 0.001), indicative of higher hydrophilicity. There were no significant differences in tensile strength (p = 0.324) for alginate and chitosan-based formulations. Preliminary testing was conducted using 0.71 μm in diameter microspheres for modeling film dissolution into Lactated Ringer’s solution. Experimental release profiles were modeled for each film from which the average release from alginate films (*M*_*AGCa*_ *= 81%*) was estimated to be twice the percentage associated with chitosan films (*M*_*CD*_ *= 42%).* The film comprised of 2.5% (w/v) medium MW chitosan/dextran 70 kDa (5:1) was selected for studying the release of 5-Fluorouracil (5-FU) as a model hydrophobic drug. Diffusion coupled with film disintegration is immediate (*t*_*B*_ = 0) in case of encapsulated 5-FU as compared to the control film encapsulating microspheres characterized by *t*_*B*_ = 70 min ± 7 min. This shift in release profile and the ability to modulate the timing of membrane burst can be attributed to the approximate ratio (1: 505) in molecular size between drug and microsphere. This hypothesis has been validated by the film pore size measured to be 430 nm ± 88 nm using atomic force microscopy.

**Electronic supplementary material:**

The online version of this article (doi:10.1186/2194-0517-2-1) contains supplementary material, which is available to authorized users.

## Background

5-fluoro-1,2,3,4-tetrahydropyrimidine-2,4-dione known as 5-Fluorouracil (5-FU) is a therapeutic agent used to treat non-melanoma skin, breast, and pancreatic cancer administered orally, intravenously (Adrucil) or topically as a cream (Effudex) (Giannola et al. 2010, 2012; Loven et al. 2002). This agent has a molecular weight of 130 g/mol, is partially soluble in water with a maximum solubility of 1 mg/ml, and has an LD_50_ = 230 mg/kg (orally in mice) (Fluorouracil 2012). As shown by the results of a recent *in vitro* study of release of 5-FU from Chitosan-Alginate microcapsules, minimal drug release has been reported in the stomach and small intestine. This is an advantage because 5-FU controlled release is required to avoid the side effects associated with such agents (Shabbear et al. 2012). Woolfson et al. reported that a 5-FU bio-adhesive patch can be used for local delivery to the uterine cervix in the condition of cervical intraepithelial neoplasia (CIN) which is common and potentially malignant, affecting women in a wide age group (Woolfson et al. 1995). There was significant delay in the absorption of 5-FU through patches due to the controlled release of drug from the patches. The half-life was statistically significant (p < 0.01) compared to intravenous route in rabbits and such half-life is observed in humans in the conventional route (Diasio & Harris 1989). The enhancement in drug delivery by the transdermal patch as compared to intravenous delivery was shown by results of a clinical evaluation of 5-FU from transdermal patches on cell-induced tumors in mice indicating a statistical increase in survival time of 7 days (p < 0.01) (Chandrashekar & Prasanth 2008). These patches were based on an ethylcellulose and polyvinylpyrrolidone K-30 (PVP K-30; ratio: 8:2) formulation, di-butylphthalate as a plasticizer, and 2% (v/w) of isopropyl myristate as a permeation enhancer. *In vitro* induction of apoptosis and cell cycle arrest by polyvinylpyrrolidone K-30 has been recently reported (Wang et al. 2003) and isopropyl myristate is classified as a skin irritant (2000).

In parallel it has been extensively documented that the use of biocompatible and biodegradable alginate and chitosan wound dressing films/patches are advantageous in many ways: 1) They kill bacteria and prevent infections caused by systemic invasion (Burkatovskaya et al. 2008); 2) They promote a better healing environment by absorbing the wound exudate (Murakami et al. 2010); 3) They eliminate the need for wiping or washing the wounds in between new dressings (Ayogi et al. 2007); 4) They have good bio-adhesive properties, eliminating the need for surgical adhesives (Khan et al. 2000). The three most common means of film drying mentioned in the above references are air drying, thermostatic, and freeze drying. Meng et al. found that the films dried in open air were drier than expected (Meng et al. 2010). Sezer et al. observed that lyophilizing resulted in thick films with larger pores (Sezer et al. 2007) and the size of the pores in the films was found to increase the concentration of chitosan, thus improving the ability of the wound dressing to absorb the wound fluids. In a more recent study of cross-linked scaffolds for tissue engineering, for the 4–12% (w/v), no change in pore size range as a function of chitosan concentration was observed; but porosity was found to be inversely proportional to the chitosan content (Jana et al. 2012).

In light of the aforementioned findings and the need for a biocompatible transdermal controlled-release mechanism for hydrophobic therapeutic agents, the specific aim of this research is to devise formulations for fabricating chitosan and alginate wound dressings with embedded fluorescent microspheres mimicking a potential drug to be encapsulated in these films. The amounts of polymers, plasticizer, and cross-linking agents used for the film formulations will be varied for this parametric study. The film will be characterized in terms of thickness, elasticity, tensile strength, sorption, and the kinetics of release will be empirically modeled to calculate the amount of compound released. Lactated Ringer solution will be used as simulated body fluid or wound exudate for wet tests. The ideal film based on the stated criteria will then be chosen to encapsulate 5-FU and studied in terms of drug release profile and pore size characterization.

## Materials and methods

### Materials

The following chemicals were purchased from Sigma-Aldrich (MO, USA): Medium MW chitosan (44887, 75-85% deacetylated), Medium MW alginic acid (A2033), Dextran 70 kDa (31390), propylene glycol (P4347), lactic acid (L6661), glycerol (G2025), calcium chloride (C5670), polyethylene glycol (81260), sodium citrate dihydrate (W302600), glacial acetic acid (320099), 5-Fluorouracil (858471). Polystyrene microspheres (Catalog No. G700B) internally- dyed with Fluorescent Green (excitation 468 nm /emission 508 nm) were purchased from Duke Scientific Corporation (CA, USA) Lactated Ringer’s solution (Catalog No 6E2323) was purchased from Baxter (IL, USA).

### Methods

The stoichiometric amounts of polymers, plasticizer, and cross-linking agents used for the film formulations used in this parametric study are outlined in Table 1.

**Table 1:** **Custom design formulations for chitosan, alginate and control films**

Film name	Polymer1	Polymer2	Plasticizer	***Crosslinking Agent***	Solvent
Chitosan	Chitosan (g)	Dextran (g)	Propylene Glycol (ml)	*Lactic acid (ml)*	DI water (ml)
CD 0.0	2.5	0.5	0	*3.0*	97.0
CD 0.5	2.5	0.5	0.5	*3.0*	96.5
CD 1.0	2.5	0.5	1.0	*3.0*	96.0
CD 1.5	2.5	0.5	1.5	*3.0*	95.5
Alginate	Alginate (g)	N/A	Glycerol (ml)	*CaCl*_*2*_ *(g)*	DI water (ml)
AGCa 0.04	2.0	-	0.4	*0.04*	99.6
AGCa 0.08	2.0	-	0.4	*0.08*	99.6
AGCa 0.12	2.0	-	0.4	*0.12*	99.6

For the chitosan films (CD) and the alginate films (AGCa), the plasticizer (propylene glycol, PG) and cross-linker (calcium chloride, CaCl_2_) concentrations were varied, respectively. Although the role of cross-linker and plasticizer differ entirely in formulating composite films, the variables were chosen to overcome the documented limitations associated with each biopolymer: addressing stiffness and lack of porosity for chitosan (Jana et al. 2012; Mobed & Chang 1998; Madsen et al. 2011), and regulation of the encapsulated compound release rate for alginate (Shabbear et al. 2012; Simpliciano & Asi 2012). Three films were fabricated for each formulation and measurements were conducted in triplicate unless indicated otherwise.

#### Film fabrication

All films were fabricated by a casting/solvent evaporation technique.

Chitosan films were fabricated based on the modification of a previously proposed formulation design, specifically with the removal of corn starch and the cytotoxic cross-linking agent glutaraldehyde from the formulation (Wittaya-arrekul & Prasharn 2006). Chitosan, dextran and 5-FU (0.07% w/v) were dispersed in DI water for 1 hr and PG was then added to this solution. This step was followed by the addition of lactic acid at a rate of 0.6 ml/min. Chitosan (pK_a_ = 6.5) is reported to form complexes with negatively-charged moieties such as sodium carboxymethylcellulose, citrates, pectin, acacia, agar, sodium caprylate, stearic acid sodium tri-polyphosphate, lactic acid, malic acid, and alginic acid (Tiwary & Rana 2010; Adusumilli & Bolton 1991; Akbuga & Bergisadi 1996; Suheyla 1997; Dureja et al. 2001; Wang et al. 2001). Although chitosan solubility is a function of molecular weight and degree of deacetylation, the reported solubility threshold for this polyelectrolyte is 4. Lactic acid (pK_a_ = 3.86) at a concentration 0.025 M acts as a solubilizer for the protonated amine group of the chitosan (Zhao et al. 2009). In this study, a 0.43 M lactic acid solution was used (3% (v/v) and thus cross-linking occurred by means of electrostatic interactions. The homogenized mixture was subsequently stirred to crosslink for 8 hrs. In the case of fluorescent microspheres (1% v/v), the particles were dispensed into the chitosan/dextran solution prior to the cross-linking step and did not undergo the pre-mixing step as is the case for 5-FU.

Alginate films were fabricated based on a modification of a previously proposed methodology (Rhim 2004). The solution was prepared by the gradual addition of alginate powder over a 1 hr period into a stirred solution of DI water, glycerol and 5-FU (0.07% w/v). Subsequently, CaCl_2_ pellets were added in one shot to cross-link the solution for 8 hrs. In the case of fluorescent microspheres (1% v/v), the particles were dispensed into the alginate/glycerol solution prior to the cross-linking step and did not undergo the pre-mixing step as is the case for 5-FU.

The stirring speed was set to 240 rpm for the dissolution and cross-linking steps for both chitosan and alginate films. This speed was chosen to overcome the gradual thickening of the polymer solutions as a result of cross-linking.

A 25 ml solution of either alginate or chitosan-based formulation was poured into a 6.06-cm diameter glass petri-dish and allowed to degas at 4°C overnight. Subsequently, the solvent (DI water) was allowed to evaporate under ambient conditions (25°C and relative humidity of 55% ± 10%) for 24 hrs. The circular dried patches were cut using scissors to have an area of approximately 10 cm^2^ and stored in an air tight container under ambient conditions for 1 week prior to use.

#### Thickness measurements

Film thickness (*th*) was measured using an EPIPHOT Nikon transmission microscope/camera (Model40) equipped with the NIS-Elements Basic Research Software imaging software. Five points were randomly taken at different locations of the film.

#### Tensile testing

The modulus of elasticity (*E*) of these wound dressings was estimated using an in-house stress–strain gage consisting of a ScoutPro scale (mg resolution) and an electronic length measurement device, the limits for which are 581.4 g and 25.75 mm, respectively. A 0.5 cm^2^ rectangular sample strip was used as test sample. The ends of films were fastened with glue to the unit, and both the scale and length measurement device were zeroed. The knob on the measurement device was then turned to a displacement of 0.5 mm and both the elongation and mass were recorded until the film fractured and the test was completed. Measurements were conducted on films encapsulating microspheres only. Displacements were applied and resultant forces were measured. Stress (σ) is subsequently calculated by dividing force by the cross-sectional area (A) of the film (Eqn. 1) (Callister & Rethwisch 2010). The strain *ε* is calculated by dividing the film displacement (elongation, ΔL) by the initial length of the stent (L) (Eqn. 2). The elastic modulus (E) is then calculated from Eqn. 3, which is the slope of the linear portion of the stress–strain curve. The tensile strength (*TS*) is also found by the stress–strain curve, which represents the maximum stress the stent can undergo under tensile stretching before the moment of necking (Eqn. 4). Tensile strength was taken as the highest point (*F*_*max*_) of the stress-train curve.

1 $$\sigma =\frac{F}{A}$$

2 $$\varepsilon =\frac{\mathrm{\Delta L}}{L_{0}}$$

3 $$E=\frac{\sigma }{\varepsilon }$$

4 $$\mathrm{TS}=\frac{F_{\max}}{A}$$

#### Sorption ability

Lactated Ringer’s solution (pH = 6.5) was used to simulate the behavior of the wound exudate or the area of lesion onto which the film might be applied (Rhim 2004). The biopolymer sorption ability was determined gravimetrically. The weights of strips of completely dried films were determined directly with a digital balance (mg resolution) and immersed into Lactated Ringer’s solution for a 24 hr period. The resultant swollen films were gently blotted with filter paper to remove excess surface water and weighed again. The sorption ability of the film is expressed in terms of percentage of weight increased using Eqn. 5.

5 $$S\left(\%\right)=\frac{W_{b}-W_{a}}{W_{b}}\;x\;100$$

where

*W*_*b*_ weight of the film before immersing in solution

*W*_*a*_ weight of the film after immersing in solution and blotting

#### Drug release testing

**Figure 1:**
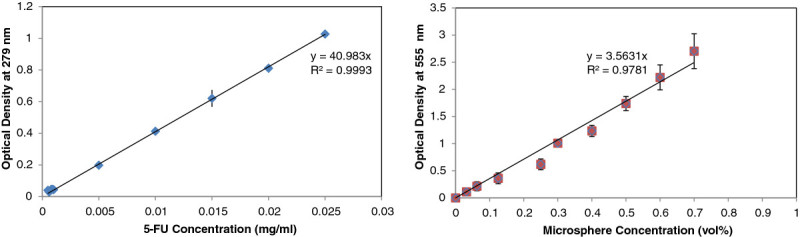
**Calibration curves for concentration determination as a function of optical density (a) 5-FU, (b) microspheres.**

At the initial stage, 5-FU release profile was simulated by the use of 0.71 *μ* m in diameter microspheres that were mixed into individual formulations and thus embedded in the films to simulate the behavior of a hydrophobic drug. Each film was immersed into a well stirred beaker simulating a mixing tank containing 100 ml of Ringer’s Lactate (pH = 6.5). Under these sink conditions, compound release occurs from all surfaces of the matrix differing from the geometry of the in-use wound dressing, a methodology pursued in previous studies (Gay et al. 1983; Tada et al. 2010). Franz cells or diffusion chambers are used in standard *in vitro* test for diffusion experiments for researching transdermal drug administration (Franz 1978; Smith & Haigh 1992). As reported, this type of testing often yields permeation data that suffer from poor reproducibility compounding the method variation with the sample non-uniformities (Ng et al. 2010; Chilcott et al. 2005). Due to the lack of knowledge on the topography, porosity, surface roughness of the films, and the associated variance, it was decided to proceed with the traditional immersion method (Gay et al. 1983; Tada et al. 2010). The release and film- dissolution profiles were characterized by analyzing the optical density in the supernatant. Samples of 0.5 ml from the supernatant mixed with 0.5 ml of Ringer’s Lactate were taken every 5 min and centrifuged at 3,000 g prior to being subjected to spectrophometric analysis using a UV–VIS spectrophotometer (Agilent 8453) at wavelengths of 555 nm for beads and 279 nm for 5-FU, respectively. Throughout the UV–VIS spectrum, the polystyrene beads will scatter light in the Mie domain (van de Hulst 1981). In addition, the size of the beads is comparable to that of bacteria for which optical density characterization due to particle light scattering is conducted in the range of 550–600 nm (Shuler & Kargi 2010) and thus the justification behind the wavelength of 555 nm for microsphere detection. Linear calibration curves for converting optical density to concentration for 5-FU and the beads are presented in Figures 1a and 1b, respectively. In order to create quantitative drug elution responses, the time release plots were compared in terms of three time periods: a) Lag phase defined as the duration of membrane hydration, b) Membrane burst phase defined as the duration of active diffusion of encapsulated substance from the films, and c) Steady-State Phase defined as the period (*dC/dt* = 0) during which the membrane has entirely disintegrated and the encapsulated substances are free in solution. The burst time (*t*_*B*_) and release time (*t*_*R*_) are defined as the duration of lag and burst phases, respectively. The duration of release per formulation as a function of time was also modeled as a function of concentration (*C)* and the steady state concentration (*C*_*SS*_). For all models residuals (SSE) were minimized using the Solver Tool in Excel 2010.

#### Pore size measurement

3 cm^2^ samples were cut from each film by a razor and glued onto a 15 mm sample disc. This disc was then placed onto the sample holder and held into place by two arms, whereupon the sample holder was placed under the atomic force microscopy (AFM) stage.

Surface imaging was performed on the CD0.5 film formulations using AFM. The characterization was conducted using an Agilent 5500 AFM using non-contact mode PPPHR-NC probes (NanoAndMore, USA). Picoview v1.8 and Gwyddion v2.3 were used as qualitative real-time and quantitative image analysis software, respectively. Experimental parameters were set in Picoview that maximized the trace/retrace profile for optimal imaging. These parameters were: scan speed of 1 line/s, resolution of 256 pixels, sample scale of 20 microns for surface roughness measurements, sample scale of 2 microns for pore size measurements, integral and proportional (I and P) gains of 10, frequency offset at −300 Hz, and stop at percentage at 95%. Four images were obtained at different locations around the sample. These locations were changed through the movement of the stage. The average pore size was obtained by taking the surface profile of the images using Gwyddion across each film. Average surface roughness (*R*_*a*_) was estimated through a statistical algorithm coded into Gwyddion.

## Results

**Figure 2:**
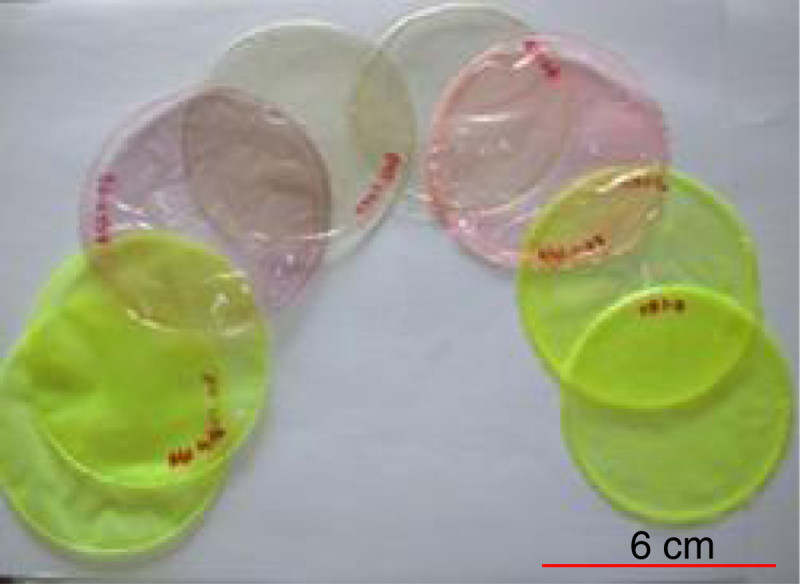
**Photograph of chitosan and alginate custom design wound dressing films.** Fluorescent microspheres are immobilized in colored specimens while the (CD0.5) encapsulating 5-FU are colorless.

Fabricated circular sample films prepared according to the formulations in Table 1, are depicted in Figure 2.

### Parametric study

**Figure 3:**
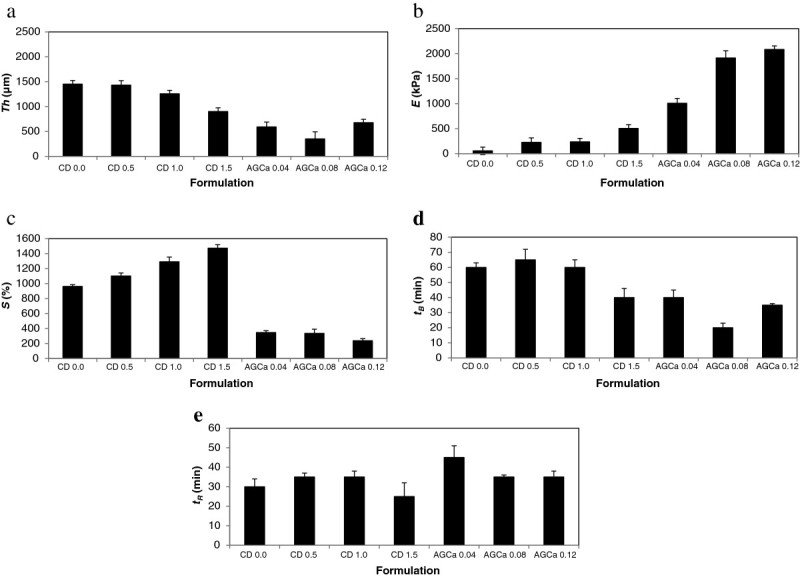
**Summary of parametric study for statistically significant film properties by formulation (a) film thickness, (b) elastic Modulus, (c) percent sorption, (d) lag phase duration,(e) duration of release.**

The summary of the parametric study encompassing the variation in film characteristics as a result of formulation is presented in Table 2 and Figures 3a-3e.

**Table 2:** **Average results of the parametric study by wound dressing film**

Film name	***Thickness (μm)***	***[E] (kPa)***	***TS (kPa)***	***S (%)***	***t***_***B***_ ***(min)***	***t***_***R***_ ***(min)***
CD 0.0	1451 ± 74	58 ± 46	18 ± 2	962 ± 24	60 ± 3	30 ± 4
CD 0.5	1431 ± 90	228 ± 40	39 ± 4	1102 ± 40	70 ± 7	50 ± 2
CD 1.0	1258 ± 68	239 ±30	64 ± 9	1293 ± 62	60 ± 5	25 ± 3
CD 1.5	901 ± 74	507 ± 80	94 ± 11	1474 ± 46	40 ± 6	35 ± 7
AGCa 0.04	592 ± 97	1010 ± 246	27 ± 8	347 ± 24	45 ± 5	55 ± 6
AGCa 0.08	351 ± 143	1915 ± 220	67 ± 7	335 ± 56	30 ± 3	35 ± 1
AGCa 0.12	677 ± 68	2086 ± 400	107 ± 13	237 ± 28	35 ± 1	35 ± 3

A one-tailed student t-test was used to compare film properties as a function of polymer type used (alginate or chitosan) at the 95% confidence interval (α = 0.05). Analysis was conducted using the Data Analysis toolbox of Excel 2010.

Alginate films are characterized by significantly higher elastic moduli (p = 0.003, *E*_*CD*_ = 258 MPa ± 185 MPa .vs. *E*_*AGCa*_ = 1670 MPa ± 578 MPa) and release windows (p = 0.03, *t*_*RCD*_ = 31 min ± 5 min .vs. *t*_*RAGCa*_ =38 min ± 6 min). A monotonic increase in tensile strength as a function of plasticizer content (PG) for the CD films and cross-linker (CaCl_2_) content for the AGCa films is observed. Chitosan films exhibit superior sorption characteristics (p = 0.001, *S*_*CD*_ = 1208% ± 233% .vs. *S*_*AGCa*_ =306% ± 60.3%), with a monotonic increase in sorption capability observed as a function of (PG) content. Chitosan films are also significantly thicker (p = 0.004, *th*_*CD*_ =1260 μm ± 255 μm .vs. *th*_*AGCa*_*=* 540 μm *±* 169 μm) as well being resistant to burst (p = 0.027, *t*_*BCD*_ = 56 min ± 11 min .vs. *t*_*BAGCa*_ = 32 min ± 10 min).

There is no significant difference in tensile strength (p = 0.324, *TS*_*CD*_ = 54 kPa ± 33 kPa .vs. *TS*_*AGCa*_ = 67 kPa ± 40 kPa) by main polymer type.

### Tensile testing

**Figure 4:**
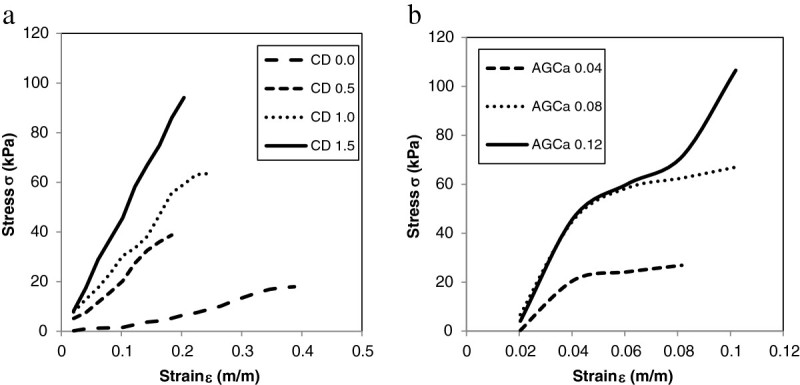
**Stress/Strain curves for wound dressings (a) chitosan films, (b) alginate films.**

Elastic modulus and tensile strength were determined per formulation from the stress strain curves displayed in Figures 4 and 4b. As mentioned above in average chitosan-based films are significantly more brittle than alginate films. Although both chitosan and alginate films display viscoelastic behaviours, chitosan has linear stress–strain curves whereas alginate has non-linear curves especially after 4% strain. Alginate films fractured at much lower strains (8-10% strain) when compared with chitosan films (18-38% strain).

### Microsphere and drug elution profiles

**Figure 5:**
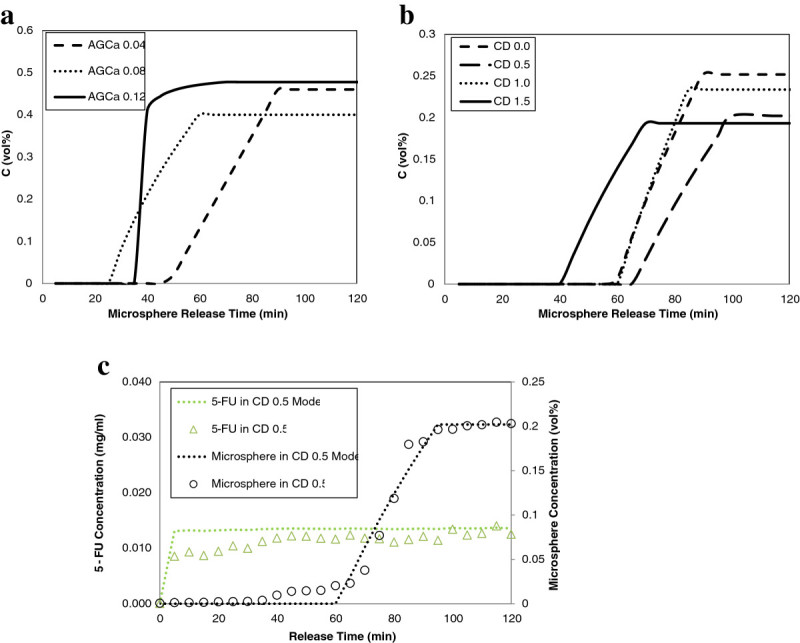
**Modeled comparative release profiles for (a) encapsulated microspheres in alginate films, (b) encapsulated microspheres and in chitosan films, (c) release of microspheres and 5-FU from CD0.5 film.**

Averaged release profiles by film formulation (N = 3) are plotted in Figures 5a and 5b for chitosan and alginate films. Corresponding empirical piecewise defined mathematical functions modeling the release of encapsulated compounds [*C(t)*] using Heaviside functions is given by Eqn. (6). Release profiles during burst phase as a function of concentration measurements [*R(t)*] are presented in Table 3 for each formulation.

6 $$C\left(t\right)=u_{t_{B}}\;\left(t\right)\;R\left(t\right)-u_{t_{R}}\left(t\right)\;\left[R\left(t\right)-C_{\mathrm{ss}}\right]$$

where

*C*(*t*) concentration of encapsulated compound in solution at anytime

*C* (*t*) = 0 *t* < *t*_*B*_

*C*(*t*) = *R*(*t*) *t*_*B*_ ≤ *t* < *t*_*R*_

*C*(*t*) = *C*_*SS*_*t* ≥ *t*_*R*_

*R*(*t*) concentration of encapsulated compound in solution during release phase (vol% or mg/ml)

*C*_*SS*_ steady state concentration of released compound in solution (vol% or mg/ml)

*t*_*B*_ membrane time to burst (min)

*t*_*R*_ duration of compound release in the supernatant (min)

$$u_{t_{B\;}}$$

Heaviside function related to *t*_*B*_

$$u_{t_{R\;}}$$

Heaviside function related to *t*_*R*_

**Table 3:** **Modeling of time release profiles from films (N = 3)**

Film	***Encapsulated compound***	***Burst phase model [R(t)]***	***C***_***SS***_	***SSE***	***M (%)***
CD 0.0	microsphere	0.606 ln (*t*) − 2.4175	0.251	0.00	49
CD 0.5	microsphere	0.4817 ln (*t*) − 2.016	0.202	0.00	41
CD 0.5	5-FU	0.025t0.20.35+t0.2	0.013	0.00	57
CD 1.0	microsphere	0.667 ln (*t*) − 2.774	0.234	0.00	46
CD 1.5	microsphere	0.355 ln (*t*) − 1.315	0.193	0.00	34
AGCa 0.04	microsphere	0.011 *t* − 0.530	0.460	0.03	99
AGCa 0.08	microsphere	0.515 ln (*t*) − 1.157	0.400	0.00	47
AGCa 0.12	microsphere	0.5t−350.80.8+t−350.8	0.477	0.06	96

As seen in Figures 5a and 5b, in accordance with the results of the statistical analysis conducted in the parametric study section above by main polymer type, chitosan films are characterized by a longer lag phase while a longer release times are associated with alginate films. The values of *t*_*B*_ ,*t*_*R*_ , *C*_*ss*_ used are average values per film and presented in Table 2. The total percentage of microsphere or drug released [M] given by Eqn. 7 can be calculated by film type from the release profile assuming the concentration of compound released in the mixing tank is uniform and the total volume of the Ringer’s solution in the tank remains constant (Saterbak et al. 2007).

7 $$M=\frac{\frac{1}{V}\int_{t_{B}}^{t_{B}+t_{R}}R'\left(t\right)\;\mathrm{dt}}{M_{0}}\;x\;100\;x\;\mathrm{DF}$$

where

*M* Percentage of encapsulated volume or mass released

*M*_0_ Initial volume fraction (ml) or amount (mg) per 100 ml

*V* Ringer’s solution volume

*DF* Dilution factor at sampling

The percentage microsphere release ranges were calculated to be 47-99% and 34%-49% for alginate and chitosan films, respectively.

Since across all formulations, the elasticity of human skin rated at 18.8 MPa (Silver et al. 2007) was not surpassed, elastic modulus was not a criterion for choosing the film formulation for studying the release of the model drug 5-FU. Chitosan, specifically the CD0.5 formulation, was chosen over alginate as the main polymer to encapsulate 5-FU because of its significantly higher sorption ability and superior resistance to membrane burst. Comparative drug elution profiles for the control CD 0.5 film encapsulating microspheres and 5-FU are presented in Figure 5c. Drug release was characterized by the absence of a lag phase (*t*_*B*_ = 0) and immediate diffusion. The percentage of 5-FU released from the CD0.5 film is 57% as compared to 41% for the CD0.5 film encapsulating the microspheres.

### AFM imaging results

**Figure 6:**
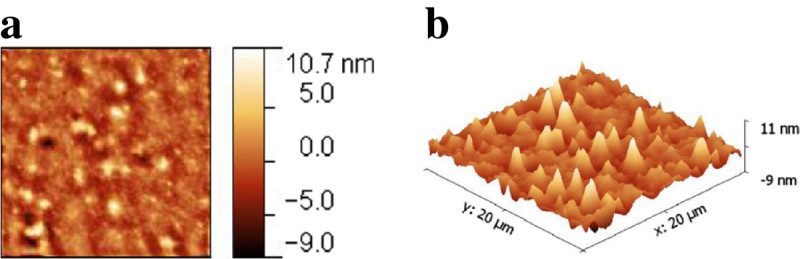
**Surface roughness determination for the CD 0.5 wound dressing film using atomic force microscopy (a) 2D view and (b) 3D view of the 20 μm** ^**2**^ **scanned area.**

**Figure 7:**
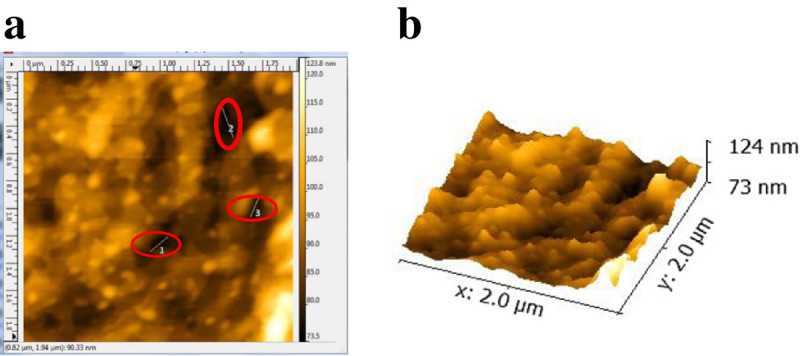
**Pore size characterization for the CD 0.5 wound dressing film using atomic force microscopy (a) 2D view where dashed circles represent the sample measured pores and (b) 3D view of the 2 μm** ^**2**^ **scanned area.**

Sample 2D and 3D views for surface roughness and pore size measurements of the CD 0.5 film based on AFM scans, are presented in Figures 6a-6b and Figures 7a-7b, respectively. The film is characterized by an average pore size of 430 nm ± 88 nm and roughness of 1.8 nm. AFM is a technique used for surface characterization and it can only be hypothesized that the recorded surface indentations are pores. However, since 5-FU diffuses through the membrane as proven by the spectrophotometric method and, membrane burst is necessary for the 710 nm microspheres to be released, it is likely that these features are pores.

## Discussion

The viscoelastic behaviors of alginate, chitosan, and alginate/chitosan have been characterized experimentally as a function of MW, blend composition, degree of substitution, and extent of cross-linking under compressive and tensile strains has been characterized by multiple sources (Storz et al. 2010; Moresi et al. 2001; Mitchell & Blanshard 1974; Torres et al. 2006; Li et al. 2012; Shon et al. 2007). As reported in the results section, for alginate films beyond 4% strains, non-linear viscoelasticity was observed. For non-blended, non-cross linked alginate gels, strains in the range of 3-10% exhibited linear viscoelastic behavior (Storz et al. 2010). Other studies showed variation of G’ (storage modulus) on frequency of oscillation and showed the dependency of viscoelastic behavior on MW and ratio of mannuronic to glucuronic groups (Moresi et al. 2001). On average, the experimental tensile strength is 1000 times lower than values previously reported for identical formulations (Rhim 2004). The reported average elongation at break of 10% coincides with the upper limit reported in this study. The root cause of this discrepancy could reside in the measurement techniques for film thickness. Their results did not indicate that as the concentration of CaCl_2_ solution increased TS increased, which is not well aligned with the positive correlation observed between the cross-linker concentration and tensile strength in this study. It could be inferred that the increase in TS and the decrease in *E* by CaCl_2_ treatment were mainly due to the development of cross-linking between carboxyl group of alginate and Ca^2+^. Correlations between G’ (storage modulus) and G” (loss modulus) as a function of concentration and oscillatory frequency have been reported on rheological studies conducted on pure chitosan (Torres et al. 2006). G’ is significantly higher than G” at low frequency oscillations and at high concentrations of chitosan indicating the predominance of elastic behavior. G” was higher than G’ at lower concentrations indicating the decrease in elastic behavior. Their lower limit of chitosan concentration corresponds to the nominal 0.025 g/mL used in this study. In another study, the tensile strength of chitosan films cross-linked with lactic acid (1.4% w/v in 1% w/v lactic acid), in absence of dextran and PG, was calculated to be 60 MPa, approximately 1000 times higher than the average measured TS for the CD films with an elongation at break of 67% (Khan et al. 2000) as compared to the measured range of 18-38%. Theoretically, in absence of the plasticizer and a lower chitosan concentration, the reported TS should have been lower; however, since the thickness of the films has not been published, it is not possible to narrow down the cause of this discrepancy.

As quantified by tensile testing measurements, alginate films were 10 times stiffer than chitosan films with a much lower fracture strain level. Chitosan is a stiff/rigid polyelectrolyte associated with conferring compressive strength to bio-membranes (Jana et al. 2012; Mobed & Chang 1998). As for the effect of plasticizer content on the reduction in thickness of the chitosan films with increase in PG content, has been reported for other hydrophilic plasticizers such as polyethylene oxide (PEO). This has been explained by the contraction of the three-dimensional film matrices due to strong molecular interactions between chitosan and PEO molecules (Li et al. 2011). In this study chitosan films are significantly thicker than alginate films and CD film thickness decreases with increasing PG content. Cited film thicknesses for chitosan wound dressings range from 0.028 to 0.13 mm (Bhuvaneshwari et al. 2011), approximately 10 times lower than the experimental values. Apart from the difference in measurement methods, it could be hypothesized that the removal of glutaraldehyde as a chemical cross-linker substituted by physical cross-linking in lactic acid, resulted in a less porous matrix, hindering evaporation. Assuming that the porosity of composite films is regulated by the amount of plasticizer (Madsen et al. 2011; Li et al. 2011), it could be inferred that porosity and hence the rate of evaporation increases with increasing PG concentrations, thus resulting in thinner CD films. As indicated by the results of tensile testing, alginate film cross-linking occurred and is dependent on cross-linker concentration. Theoretically, an expected monotonous decrease in film thickness as a function of degree of cross-linking, and thus higher porosity, should have been recorded. It could be inferred that the experimental range for the CaCl_2_ is this study is not large enough to trigger significant porosity changes.

In this study the chitosan films exhibited significantly higher hydrophilicity and thus a higher affinity for wound exudate simulated by the Lactated Ringer’s solution. The results of the sorption ability of the films indicate a growing hydrophilicity with an increase in PG concentration in chitosan films in agreement with previous findings (Ayogi et al. 2007; Wittaya-arrekul & Prasharn 2006) identical to the behavior of PEGylated chitosan derivatives (Bhuvaneshwari et al. 2011). The sorption values of the films are on average 1–5 times higher than the values previously reported (Wittaya-arrekul & Prasharn 2006) due to the higher film thicknesses measured in this study. As the degree of cross-linking increases, alginate water solubility decreases, resulting in lower sorption capability of the alginate films (Rhim 2004).

Atomic force microscopy was used in previous studies looking at the surface roughness and pore size of chitosan and chitosan alginate composite films (Casettaria et al. 2012; Dash et al. 2011; Hu et al. 2007; Karakecili et al. 2007; Zheng et al. 2009; Doulabia et al. 2013). It has been reported that the surface topography of 100% chitosan had a smooth surface with uniformly distributed short spikes, but when an additive was introduced, the surface roughness increased resulting in taller spikes (Doulabia et al. 2013). In these articles the *R*_*a*_ for several formulations/methods of chitosan film fabrication ranges from 0.3- to 4.6 nm (Casettaria et al. 2012; Dash et al. 2011; Hu et al. 2007). The experimental value of 1.8 nm falls within this reported range. The measured pore sizes of 433 nm ± 88 nm are larger than the 2.8 -100 nm previously reported for a scan area of 5 μm^2^ as compared to the 2 μm^2^ adopted in this study (Hu et al. 2007).

It could be inferred that chitosan rigidity, in addition to thicker-wound dressing walls, are the driving forces behind the statistically significantly higher average membrane burst time as compared to alginate films. Even after membrane burst, the average release from alginate films (*M*_*AGCa*_ = 81%) is approximately twice that calculated from chitosan films (*M*_*CD*_ = 42%) from which, it could be inferred that alginate films are more porous than chitosan films and hence, the justification of using chitosan for slowing down the release rate of compounds from alginate membranes (Gaserod et al. 1999; Asthana et al. 2012). Theoretically, an increase in CaCl_2_ for the alginate films in conjunction with an increase in PG content for the chitosan films, should increase the porosity of the films, and hence the diffusion rate (Madsen et al. 2011; Simpliciano & Asi 2012); however, no discernible trends were observed as a result of varying the aforementioned factors. These observations are limited by the unknown distribution of surface-to-through pores for each film obtainable through scanning electron microscopy.

As previously stated, 5-FU release from the CD 0.5 film was characterized by the absence of a lag phase as compared to the microsphere release profile from the same control chitosan-based film. 5-FU has a Stokes radius of 0.372 nm Fournier (2011) as compared to the 710 nm microsphere. Hence, the absence of lag phase for the 5-FU release profiles is attributed attributed to the approximate ratio (1: 505) in molecular size. Given the film pore size of 430 nm ± 88 nm, membrane burst due to osmosis is not necessary for drug diffusion while it is for the larger microspheres. Identical release profiles as in Figure 5c were generated for the co-encapsulation of the drug and the microsphere, demonstrating the immediate diffusion of the drug followed by membrane burst releasing the microspheres (Rook et al. 2012). This two stage release has been recently documented for the release of curcumin co-encapsulated with silica microspheres in chitosan scaffolds (Ahmed et al. 2012) although the mechanism of burst has not been elaborated upon. Revisiting the formulation of the 5-FU film in order to restore the lag phase into the elution profile and thus modulate membrane burst time, the drug should be first encapsulated into microspheres/nanoparticles then immobilized within the wound dressing film, a successful approach adopted to modulate drug release and minimize the membrane burst effects (Tada et al. 2010; Ramadas et al. 2000; Dhoot & Wheatley 2003).

## Conclusion and future efforts

Composite films of chitosan and alginate, intended for drug delivery/wound healing applications, were fabricated and characterized. It was revealed by this *in-vitro* evaluation that alginate films are significantly stiffer. Meanwhile chitosan films are thicker, more resistant to osmotic burst and are more hydrophilic as characterized sorption rates. With the successful elimination of glutaraldehyde from the chitosan film formulations, 5-FU was encapsulated as a model drug into a chitosan film (CD 0.5) comprised of 2.5% (w/v) Medium MW chitosan/dextran 70 kDa (5:1) using 0.5 and 3% (v/v) of PG and lactic acid, respectively. The translated microsphere release profiles modeled using Heaviside functions as compared to 5-FU release characterized by the absence of a lag phase, are supported by AFM pore size measurements. Future porosity characterization should encompass scanning electron microscopy (SEM) measurements to determine the nature and directionality of the pores as well as the distribution of surface-to- through pores. Also, at a known porosity and nominal film thickness, fluorescent spatial and temporal concentration gradients should be measured in order to obtain diffusivity coefficients for optimization of the desired pharmacokinetic flux. *In-vitro* cytotoxicity testing will be added to the protocol to assess the effect of the glutaraldehyde removal from the chitosan formulations.

## Authors’ information

RKN: Is a recent graduate of the Biomedical Devices graduate program at SJSU with a Bachelor’s Degree in Medical Electronics. SLR: Is a recent graduate of the Biomedical Devices Graduate program at SJSU with a Bachelor’s Degree in Chemical Engineering. JJR: Is a graduate student in the Biomedical Engineering program at SJSU with with a Bachelor’s Degree in Chemical Engineering. MK: Dr. Keralapura is an Assistant Professor in the department of Electrical Engineering and Director of the Biomedical Systems Laboratory at SJSU. She has extensive experience in fundamental mechanical measurements of tissue and tissue-like media using both rheological techniques with ultrasound strain imaging. She is a highly respected young contributor in the field of Medical Physics. MG: Dr. Goedert is an Adjunct Professor in the department of Biomedical, Chemical and Materials Engineering at SJSU and a French Chemistry Society Prize winner. He has over 20 years of experience in high precision sensor development and 10 years of experience in micro-channel surface characterization. MMM: Dr. Mobed-Miremadi is an instructor and Endowed Chair of Bioengineering at SJSU. She has researched microencapsulation methods extensively and has over 10 years of experience in the biomedical industry specifically in the areas of inkjet bio-printing .bio-reactor design genomics (micro-array fabrication) and medical devices.

## Authors’ original submitted files for images

Below are the links to the authors’ original submitted files for images. Authors’ original file for figure 1 Authors’ original file for figure 2 Authors’ original file for figure 3 Authors’ original file for figure 4 Authors’ original file for figure 5 Authors’ original file for figure 6 Authors’ original file for figure 7

## Abbreviations

5-FU: 5-fluoro-1,2,3,4-tetrahydropyrimidine-2,4-dione
CD: Chitosan-dextran film formulation
AGCa: Alginate film formulation
*th*: Film thickness (μm)
*E*: Elastic modulus (Pa)
*TS*: Tensile strength (Pa)
*S*: Sorption ability (%)
*t*_*B*_: Membrane time to burst or lag phase duration (min)
*t*_*R*_: Duration of release from membrane or release window5-FU (min)
*M*: Percentage of encapsulated mass or volume released.
