# Supplementary figures and images for: Polystyrene microsphere and 5-fluorouracil release from custom-designed wound dressing films
Source: Prog Biomater. 2013 Jan 24;2:1. doi: 10.1186/2194-0517-2-1 (PMC5151105; doi:10.1186/2194-0517-2-1)

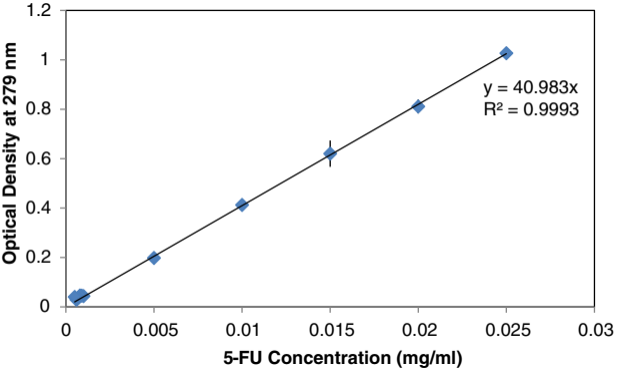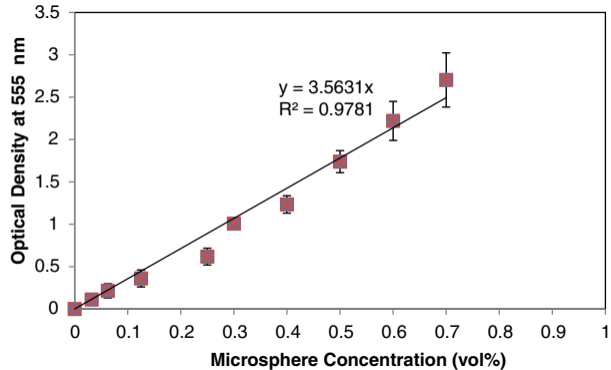

Supplement: Supplementary file 1 — Authors’ original file for figure 1 [file 40204_2012_6_MOESM1_ESM.pdf]

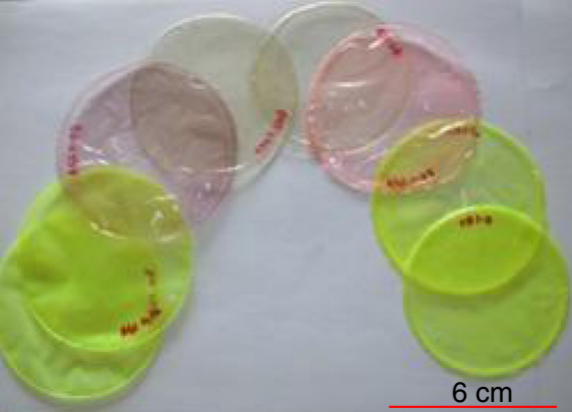

6 cm

Supplement: Supplementary file 2 — Authors’ original file for figure 2 [file 40204_2012_6_MOESM2_ESM.pdf]

**a**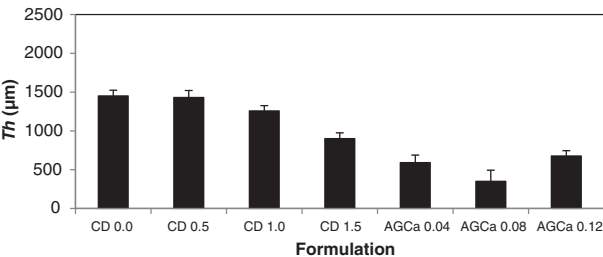**b**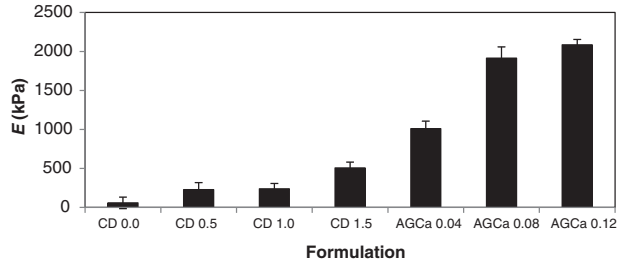**c**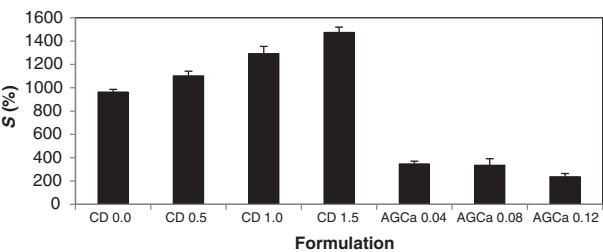**d**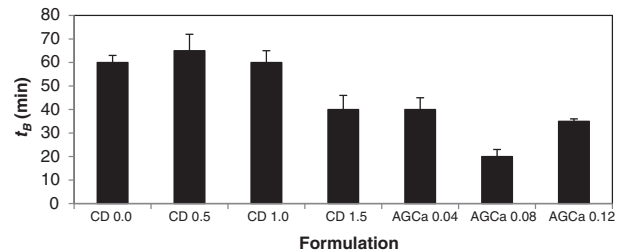**e**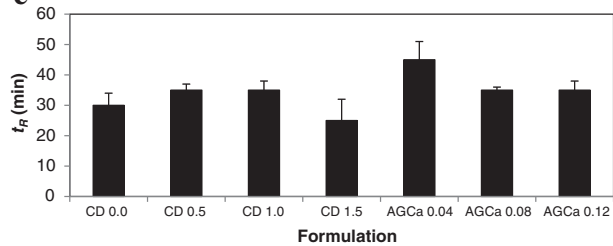

Supplement: Supplementary file 3 — Authors’ original file for figure 3 [file 40204_2012_6_MOESM3_ESM.pdf]

a

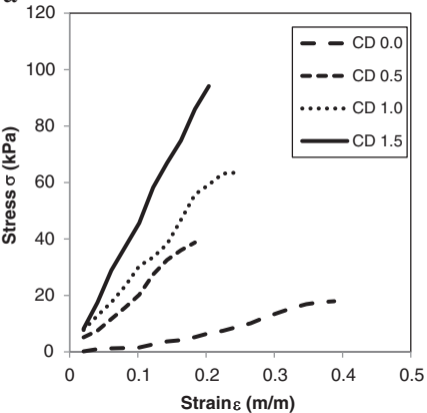

b

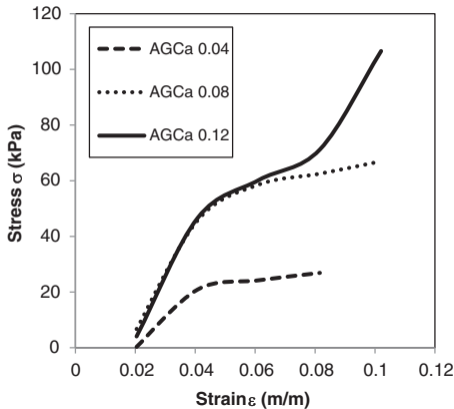

Supplement: Supplementary file 4 — Authors’ original file for figure 4 [file 40204_2012_6_MOESM4_ESM.pdf]

**a**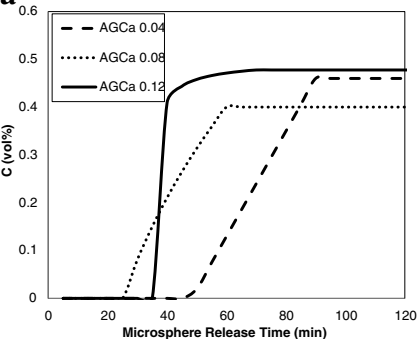**b**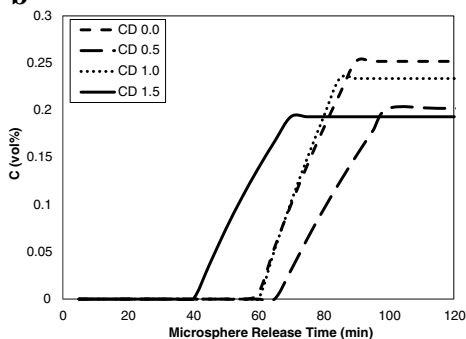**c**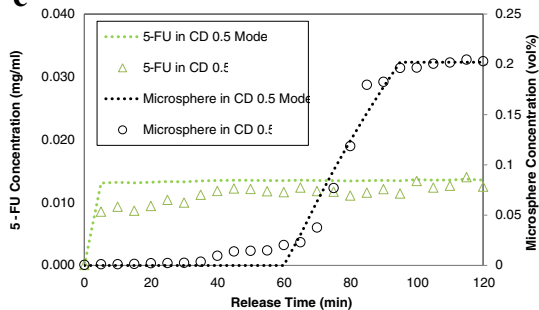

Supplement: Supplementary file 5 — Authors’ original file for figure 5 [file 40204_2012_6_MOESM5_ESM.pdf]

**a**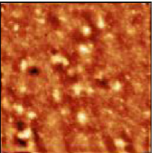**b**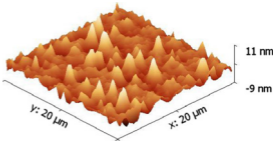

Supplement: Supplementary file 6 — Authors’ original file for figure 6 [file 40204_2012_6_MOESM6_ESM.pdf]

**a**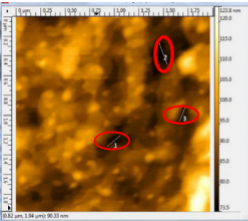**b**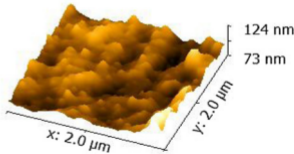

Supplement: Supplementary file 7 — Authors’ original file for figure 7 [file 40204_2012_6_MOESM7_ESM.pdf]
